# Effect of the Aged Garlic Extract on Cardiovascular Function in Metabolic Syndrome Rats

**DOI:** 10.3390/molecules21111425

**Published:** 2016-10-26

**Authors:** Israel Pérez-Torres, Juan Carlos Torres-Narváez, José Pedraza-Chaverri, María Esther Rubio-Ruiz, Eulises Díaz-Díaz, Leonardo del Valle-Mondragón, Raúl Martínez-Memije, Elvira Varela López, Verónica Guarner-Lans

**Affiliations:** 1 Department of Pathology, Instituto Nacional de Cardiología “Ignacio Chávez”, Juan Badiano 1, Sección XVI, Tlalpan, Mexico City 14080, Mexico; pertorisr@yahoo.com.mx; 2 Department of Pharmacology, Instituto Nacional de Cardiología “Ignacio Chávez”, Juan Badiano 1, Sección XVI, Tlalpan, Mexico City 14080, Mexico; juancarlostn63@hotmail.com (J.C.T.-N.); leonardodvm65@hotmail.com (L.d.V.-M.); 3 Facultad de Química, Department of Biology, Edificio B, Segundo Piso, Laboratorio 209, Universidad Nacional Autónoma de México (UNAM), Ciudad Universitaria, Mexico City 04510, Mexico; pedraza@unam.mx; 4 Department of Physiology, Instituto Nacional de Cardiología “Ignacio Chávez”, Juan Badiano 1, Sección XVI, Tlalpan, Mexico City 14080, Mexico; esther_rubio_ruiz@yahoo.com (M.E.R.-R.); varelopz@yahoo.com (E.V.L.); 5 Department of Reproductive Biology, Instituto Nacional de Ciencias Médicas y Nutrición “Salvador Zubirán”, Vasco de Quiroga 15, Sección XVI, Tlalpan, Mexico City 14000, Mexico; eulisesd@yahoo.com; 6 Electromechanical Instrumentation, Instituto Nacional de Cardiología “Ignacio Chávez”, Juan Badiano 1, Sección XVI, Tlalpan, Mexico City 14080, Mexico; raulmmemije@yahoo.com

**Keywords:** aged garlic extract, metabolic syndrome, cardiovascular functioning, oxidative stress

## Abstract

The antioxidant properties of aged garlic extract (AGE) on cardiovascular functioning (CF) in metabolic syndrome (MS) remains poorly studied. Here we study the AGE effects on CF in a rat model of MS. Control rats plus saline solution (C + SS), MS rats (30% sucrose in drinking water from weaning) plus saline solution (MS + SS), control rats receiving AGE (C + AGE 125 mg/Kg/12 h) and MS rats with AGE (MS + AGE) were studied. MS + SS had increased triglycerides, systolic blood pressure, insulin, leptin, HOMA index, and advanced glycation end products. AGE returned their levels to control values (*p* < 0.01). Cholesterol was decreased by AGE (*p* = 0.05). Glutathion and GPx activity were reduced in MS + SS rats and increased with AGE (*p* = 0.05). Lipid peroxidation was increased in MS + SS and AGE reduced it (*p* = 0.001). Vascular functioning was deteriorated by MS (increased vasocontraction and reduced vasodilation) and AGE improved it (*p* = 0.001). Coronary vascular resistance was increased in MS rats and AGE decreased it (*p* = 0.001). Cardiac performance was not modified by MS but AGE increased it. NO measured in the perfusate liquid from the heart and serum citrulline, nitrites/nitrates were decreased in MS and AGE increased them (*p* < 0.01). In conclusion, AGE reduces MS-induced cardiovascular risk, through its anti-oxidant properties.

## 1. Introduction

The risk of cardiovascular disease (CVD) increases in metabolic diseases which have a high prevalence in modern societies such as obesity, metabolic syndrome (MS), and/or type-2 diabetes mellitus [1]. Metabolic diseases alter autonomic regulation of blood pressure, glucose homeostasis, and insulin secretion [1]. Oxidative stress is also increased in the endothelium of subjects suffering from these diseases, favoring increased blood pressure, and other CVD [2]. Mitochondrial and extra-mitochondrial production of reactive oxygen species (ROS) and reduced antioxidant defense mechanisms are characteristic of the myocardium and vessels of humans and animals with the MS [3]. Increased glucose and insulin levels also modify endothelin secretion and arterial vasoreactivity [4]. The knowledge of the molecular basis for the association of obesity, MS and CVD is required to prevent its development. Thus, the possible use of natural compounds acting upon these mechanisms, including oxidative stress, is of great importance.

Extracts of raw garlic soaked in aqueous ethanol under ambient environments for over four months or longer, known as aged garlic extracts (AGE), contain phytochemicals such as water and lipid soluble organosulfur compounds, allicin, and selenium which protect against oxidative damage [5]. This treatment of raw garlic eliminates the majority of its irritating taste and odor compounds and renders it less toxic [5,6]. AGE has less secondary harmful effects than the administration of raw garlic including the decrease in erythrocytes, the increase in reticulocytes, and generation of papilloma in the fore stomach [7].

AGE might be helpful for the treatment of diseases mediated by ROS such as the MS-induced CVD [8]. In AGE, compounds with antioxidant properties such as allicin, *S*-allylcysteine, and *S*-allylmercaptocysteine are stabilized [5]. These stable compounds exert antioxidant action by scavenging ROS; increasing the activity of cellular antioxidant enzymes such as superoxide dismutase, catalase, and glutathione peroxidase (GPx); and increasing glutathione (GSH) in the cells [5]. AGE inhibits lipid peroxidation (LPO), reduces ischemic/reperfusion damage and inhibits oxidative modification of LDL, thus protecting endothelial cells from the injury by the oxidized molecules [5].

Garlic has been widely used to treat cardiovascular problems and the effects of raw garlic and AGE upon cardiovascular functioning are well documented. In a revision by Varshney and Budoff [9] on garlic supplementation, in which four meta-analyses and two original studies were analyzed, it was found that it reduces systolic blood pressure (SBP) by 7–16 mm Hg and diastolic blood pressure by 5–9 mm Hg. Garlic supplementation also reduced total cholesterol by 27.4–29.8 mg/dL in eight meta-analyses. AGE had more consistent benefits than raw garlic [9]. Rare adverse reactions were documented with limited established causality.

Although the effect of garlic and AGE has been widely established in CVD, there are only few reports on the effect of AGE on MS, diabetes, and MS-related cardiovascular diseases. There are even reports stating that the treatment with AGE had no significant effect upon metabolic parameters including insulin resistance (IR) in subjects with type-2 diabetes at risk of developing CVD [10]. In that study, treatment with AGE had no significant effect on markers of endothelial function, oxidative stress or inflammation [11]. However, the antioxidant properties of AGE might protect against oxidative stress-induced damage in the vascular endothelium and myocardium in subjects with MS [9,11]. AGE has not only antioxidant properties but it also contains selenium which might increase the activity of selenium depending enzymes such as GPx, whose activity is decreased in plasma, arteries, and hearts of subjects with MS [12].

Due to the lack of reports on the antioxidant properties of AGE on cardiovascular functioning in subjects with MS and of the contradictory results reported in subjects with type-2 diabetes at risk of developing CVD, in this paper we study the protective effects of AGE on the cardiovascular function in a rat model with MS. The MS is induced in these rats by a high ingestion of sucrose (30%) in drinking water for 24 weeks.

## 2. Results

### 2.1. Effect of AGE on the Cluster of Risk Factors That Constitute MS

Rats ingesting the high sucrose diet and receiving SS (MS + SS) exhibited more than three of the signs that constitute the cluster of MS; namely they had significantly increased levels of TG, SBP, insulin, HOMA index, leptin, and AGEs in comparison with C + SS and MS + AGE (*p* < 0.05, *p* = 0.01 and *p* = 0.001 respectively, Table 1). Values were similar in C + SS and C + AGE rats.

Cholesterol did not show significant changes in C + SS and MS + SS, but was decreased by AGE treatment in MS + AGE rats and C + AGE rats (*p* = 0.01, Table 1). Glucose did not show significant changes in any group.

To understand the changes in vascular reactivity and heart function, NO and some of its metabolites were determined. NO was measured in the liquid with which the heart was perfused and it was significantly decreased in the MS + SS rats in comparison to C + SS (*p* = 0.01). AGE treatment significantly increased NO levels in both C + AGE and MS + AGE rats in comparison with C + SS and MS + SS (*p* = 0.001, Table 1). Citrulline, a metabolite of eNOS activity, remained unchanged in the C + SS and C + AGE rats; however, its levels were decreased in MS + SS rats in comparison to C + SS and MS + AGE (*p* = 0.001, Table 1). The NO_3_^−^/NO_2_^−^ ratio, which is inversely proportional to oxidative damage, was similar in C + SS and C + AGE rats. It was diminished in MS + SS rats and returned to normal values with the AGE treatment in MS + AGE rats (*p* = 0.01, Table 1).

### 2.2. Effect of AGE on Heart Functioning and Vascular Reactivity

**Figure 1:**
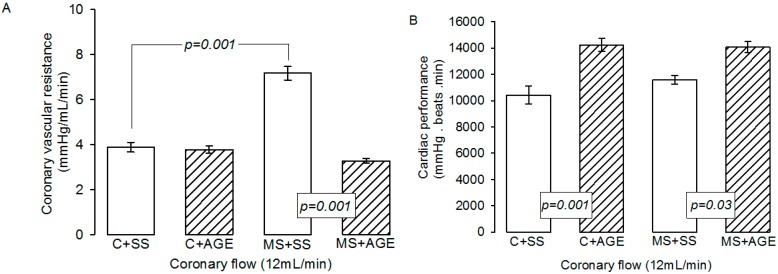
Coronary vascular resistance (**A**) and cardiac performance (**B**) in the experimental groups: Control rats plus saline solution (C + SS), MS rats (30% sucrose in drinking water from weaning) plus saline solution (MS + SS), control rats receiving AGE (C + AGE), and MS rats with AGE (MS + AGE) C + SS. Coronary vascular resistance increased in MS and the garlic treatment restored it to its control values. Cardiac performance was not modified by MS but garlic treatment increased it in C and MS. Data show the mean ± SEM, *n* = 8.

The CVR was significantly increased in MS + SS rats in comparison to C + SS (*p* = 0.001). The AGE treatment did not modify it in C + AGE rats, but decreased it in MS + AGE rats (*p* = 0.001, Figure 1A). Cardiac performance was not different in C + SS and MS + SS, but AGE treatment significantly increased it in C + AGE and MS + AGE rats (*p* = 0.001 and *p* = 0.03 respectively, Figure 1B). Furthermore, in isolated hearts of C + AGE and MS + AGE rats we observed an improvement in cardiac performance of 23% and 17%, respectively (Figure 1B).

**Figure 2:**
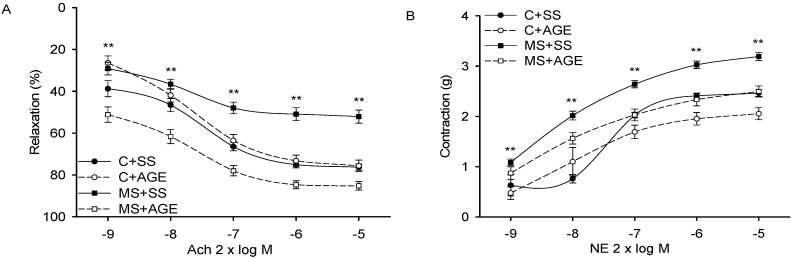
Aortic relaxation by acetylcholine in norepinephrine pre contracted arteries (**A**) and contraction by NE (**B**) in the experimental groups: Control rats plus saline solution (C + SS), MS rats (30% sucrose in drinking water from weaning) plus saline solution (MS + SS), control rats receiving AGE (C + AGE), and MS rats with AGE (MS + AGE) C + SS. Relaxation was reduced and contraction increased by MS and the AGE treatment restored vascular functioning. ** C + SS and MS + AGE vs. MS + SS *p* = 0.001. Data show the mean ± SEM, *n* = 8.

Vascular relaxation by Ach in aortic rings pre-contracted with NE, was significantly decreased in MS + SS when compared to that of C + SS (*p* = 0.001). It was similar in C + SS and C + AGE rats, but in MS rats it was significantly increased by the AGE treatment when compared to the MS + SS rats (*p* = 0.001, Figure 2A). Vasoconstriction of aortic rings stimulated by NE was significantly increased in MS + SS when compared to the C + SS rats (*p* = 0.001), but in MS + AGE rats it was decreased in comparison to MS + SS rats (*p* = 0.001, Figure 2B). In C + SS and C + AGE rat aortas, vasoconstriction was similar.

### 2.3. Glutathione Peroxidase Activity and Glutathione Concentration

**Figure 3:**
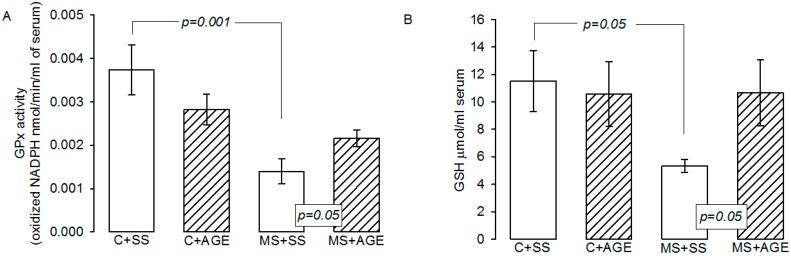
GPx activity (**A**) and glutathion levels (**B**) in the experimental groups: Control rats plus saline solution (C + SS), MS rats (30% sucrose in drinking water from weaning) plus saline solution (MS + SS), control rats receiving AGE (C + AGE), and MS rats with AGE (MS + AGE) C + SS. GPx activity and glutathion levels were decreased by MS and the AGE treatment restored them. Data show the mean ± SEM, *n* = 8.

The GPx activity was significantly diminished in MS + SS rats in comparison to C + SS (*p* = 0.001) and increased with the AGE treatment (*p* = 0.05). AGE treatment did not modify GPx activity in C + SS rats (Figure 3A). GSH was decreased in rats with MS + SS in comparison with C + SS, and AGE treatment in MS rats restored GSH levels (*p* = 0.05, Figure 3B).

**Figure 4:**
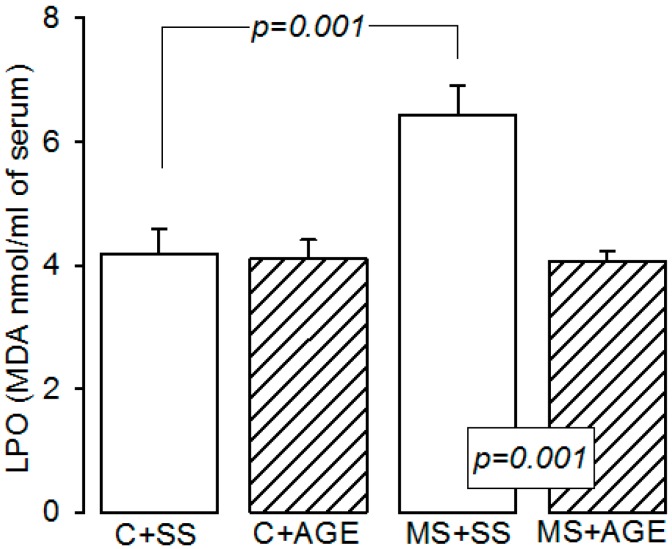
Lipid peroxidation measured by malondialdehyde levels in the experimental groups: Control rats plus saline solution (C + SS), MS rats (30% sucrose in drinking water from weaning) plus saline solution (MS + SS), control rats receiving AGE (C + AGE), and MS rats with AGE (MS + AGE) C + SS. Lipoperoxidation was increased in MS and the AGE treatment diminished it. Data show the mean ± SEM, *n* = 8.

Figure 4 shows the malondialdehyde levels as an indicator of LPO. We observed that, in serum from the MS + SS group, there was a higher level than in the C + SS group. There was a significant decrease in malondialdehyde concentration in MS rats treated with AGE (*p* = 0.001), while in C group this parameter remained constant.

## 3. Materials and Methods

### 3.1. Animals

Experiments in animals were approved by the Laboratory Animal Care Committee of our institution and were conducted in compliance with the Guide for the Care and Use of Laboratory Animals of the NIH. Weanling male Wistar rats weighing 100 ± 5 g (*n* = 8 per group) were separated into two groups: group 1, control rats (C), given tap water for drinking; and group 2, MS rats, receiving 30% sugar in their drinking water for 24 weeks. At the end of the 24 weeks, the weight of control rats was of 502 + 19.5 g and of rats receiving sucrose of 515 + 13.4 g. A half of each group of rats (control or MS) was injected intra-abdominally daily every 12 h for one month with either, saline solution as a vehicle, or 125 mg/kg/12 h of AGE (Kyolic^®^ liquid, dietary supplement; Wakunaga of America Co Ltd., Mission Viejo, CA, USA) [13]. Kyolic^®^ formula has been previously analyzed by standard high-performance liquid chromatography (HPLC) procedure for content of garlic sulfur-containing compounds (alliin,-glutamyl-Sallylcysteine and -glutamyl-1-propenylcysteine) by other authors [14]. MS rats continued receiving sucrose during the AGE treatment. Thus, the experimental groups were: (a) control rats plus saline solution (C + SS); (b) MS plus saline solution (MS + SS); (c) C plus aged garlic extract (C + AGE); and (d) MS plus AGE (MS + AGE).

The animals were housed in ad hoc plastic boxes and were subjected to 12 h light/obscurity cycle and environmental temperature between 18 and 26 °C. They were fed commercial rodent pellets (PMI Nutrition International, Inc., LabDiet 5008, Richmond, IN, USA) ad libitum. At the end of the experimental period, the mean blood pressure was determined by a plethysmographic method [3].

### 3.2. Biochemical Variables

Determination of some serum biochemical variables from the rats, such as glucose, cholesterol, triglycerides (TG), leptin, advanced glycation end products, and insulin were made by ELISA using commercially obtained kits. The HOMA index of resistance to insulin was calculated. HOMA-IR = (Insulin μU/mL) × (Glucose mM)/22.5.

### 3.3. Nitric Oxide Quantification

Quantification of NO was carried out in the perfusate liquid from the heart according to methods previously by Tenorio et al. [15] by a spectrophotometric method in UV-Vis region (UV-visible spectrophotometer (Cary 4000, Varian Inc., Mulgrave, Victoria, Australia) to 572 nm and 587 nm, at room temperature. Results were expressed in pmoles/mL.

### 3.4. Citrulline Determination

100 µL of serum were incubated for 30 min at 37 °C with 50 µL urease 12 mg/mL. 3 mL of chromic mixture were added. The chromic mixture consisted of 25% H_2_SO_4_, 20% H_3_PO_4_, 9.24 µM FeCl_3_.6H_2_O, 0.125% 2,3-butanedione monoxime, and 0.0075% thiosemicarbazide mixed by vortexing and incubated at 100 °C for 5 min. The samples were cooled to room temperature and the color developed was measured at 530 nm. The calibration curve was made with a standard solution of l-citrulline 1 µmol/L from Sigma-Aldrich (St. Louis, MO, USA).

### 3.5. Nitrate and Nitrite Quantification

100 µL of serum were incubated with 50 µL Cu-Cd for 30 min. The mixture was centrifuged an 850 g at room temperature and the supernatant was recovered. 100 µL of 10% ZnSO_4_ and 100 µL of 0.5 N NaOH were added to the supernatant and centrifuged at 7155 g. The supernatant was recovered and incubated in the presence of 200 µL of Griess reagent (100 µL 1% sulphanilamide and 100 µL 0.1% *N*-napthyl-ethylenediamine). The total volume was adjusted to 1 mL. A calibration curve was obtained with solutions of KNO_2_ ranging from 5 nmol/mL to 0.156 nmol/mL. Absorbance was measured at 540 nm.

### 3.6. Isolated Heart Perfused by the Langendorff Method

The animals were anesthetized with sodium pentobarbital (60 mg/kg body weight) and anticoagulated with heparin (1000 U/mL/kg body weight). Through a thoracotomy, the heart was exposed and the ascending aorta was referred with a silk thread. The heart was then quickly removed, placed in isotonic saline at 4 °C, and immediately connected to the perfusion system through the ascending aorta [16]. Once the heart was connected, it was given an adaptation period of 30 min to the new perfusion condition (5 min with a flow of 25 mL/min and 25 min with a flow of 12 mL/min). Heart rate (HR) was maintained at 312–324 beats per minute, by the use of a Grass stimulator (U7, Grass Instruments Co., Quincy, MA, USA). Coronary flow (F) was held at 12 mL/min with a peristaltic pump (SAD22, Grass Instruments Co., Quincy, MA, USA) throughout the experimental period. During all experiments, parameters such as left intraventricular pressure (LIVP) were recorded by using a latex balloon attached to a Grass hydroneumatic pressure transducer. The balloon was placed by advancing a catheter through the mitral valve into the left ventricle and once inside the cavity, an internal pressure of 5–10 mmHg (diastolic pressure) was applied. Perfusion pressure (PP) was also recorded and a range of 30–50 mmHg at the start of the experiment was considered as an inclusion criterion. With the values of HR and LIVP, cardiac mechanical performance (CMP) was calculated as HR × LIVP = CMP. Coronary vascular resistance (CVR) was calculated using the following relationship: PP/F = RVC [17]. After the adaptation time, liquid effluent samples were taken every 10 min for 30 min. In these samples, we measured indirectly the nitric oxide (NO) levels.

After the heart extraction the blood was collected. The samples were centrifuged for 20 min at 936 g and 4 °C, in order to collect the serum in aliquots of 400 μL and stored at −70 °C until used.

### 3.7. Vascular Reactivity

After the heart extraction and blood collection, the thoracic aorta was dissected and vascular reactivity was investigated in sections 2 mm of aortic rings, which were placed in glass chambers with Krebs solution, according to methods previously by Pérez-Torres et al. [3].The aortic ring preparations were incubated with 2 × 10^−9^ to 2 × 10^−5^ M of norepinephrine (NE) to evaluate vascular contraction The vasodilator activity was determined by cumulative concentration response curves to acetylcholine (Ach, 2 × 10^−9^ to 2 × 10^−5^ M) on NE (2 × 10^−7^) in pre-contracted aortic rings.

### 3.8. Glutathione Peroxidase Activity

100 μL of serum were suspended in 1.6 mL of 50 mM phosphate buffer (pH 7.3), to which 0.2 mM NADPH, 1 mM GSH and 1 IU mL glutathione reductase were added. The mixture was incubated for 3 min at 37 °C, then 100μL of 0.25 mM H_2_O_2_ were added to start the reaction and the absorbance was monitored for 10 min at 340 nm [18]. Activity is expressed in µmol of oxidized NADPH/min/mL serum.

### 3.9. Determination of GSH Concentration

To determine GSH concentration, 800 μL of phosphate buffer 50 mM, pH 7.3, plus 100 μL of Ellmanreactive (5,5′-dithiobis-2-nitrobenzoic) 1 M were added to 100 μL of serum previously deproteinized with 20% trichloroacetic acid (*v*/*v*) and centrifuged to 1560 g for 5 min. The mixture was incubated at room temperature for 5 min and absorbance was read at 412 nm. The calibration curve was done with GSH at concentrations from 5 to 25 μmol/mL [18].

### 3.10. Lipid Peroxidation Levels

50 μL CH_3_-OH with 4% BHT plus phosphate buffer pH 7.4 were added to 100 μL of serum. The mixture was shaken vigorously in vortex for 5 sand then incubated in a water bath at 37 °C for 30 min. 1.5 mL of 0.8 M thiobarbituric acid were then added and the sample was then incubated in a water bath at boiling temperature for 1 h. After this time and to stop the reaction, the samples were placed on ice; 1 mL 5% KCl was added to each sample as well as 4 mL *n*-butanol. They were shaken in vortex for 30 s and centrifuged at 1500 g at room temperature for 2 min. Then the *n*-butanol phase was extracted and the absorbance was measured at 532 nm. The calibration curve was obtained using tetraethoxypropane as standard [18].

### 3.11. Statistical Analysis

Statistical analysis and graphics were performed with a SigmaPlot 12.3 program (version 2016, Systat Software Inc., San Jose, CA, USA). The data are presented as the mean ± SEM. Statistical significance was determined by one-way ANOVA test, followed by the post-hoc Tukey test. Differences were considered statistically significant when *p* < 0.05.

## 4. Discussion

Although evidence has accumulated on the antioxidant effects of AGE [5] and it is known that oxidative stress underlies the increased risk of CVD associated to MS [19], there are few reports supporting the effect of garlic upon the diverse pathologies clustered in MS and their cardiovascular consequences [10]. Therefore, in this paper, we study the effects of AGE on vascular reactivity, heart function, and the altered variables comprising MS, in a rat model induced by the ingestion of 30% sucrose for 24 weeks. This model of MS has been extensively studied by our group [3,4] and mimics the high ingestion of sucrose in many populations through the drinking of soda beverages. The disease takes 24 weeks in being established and thus the study is performed in relatively aged animals, in contrast to studies done in young adults. Although cardiovascular events in MS are appearing at younger ages, many of the cardiovascular consequences of MS happen in individuals who have outrun young adulthood [20]. Therefore, the study of populations of this age in the rat is important, since it might be similar to that of patients suffering from the cardiovascular consequences of MS.

In our study, rats developed MS having at least three of the criteria used to diagnose the disease. They were hypertriglyceridemic and had hypertension and IR. This data are in accordance with those previously published by our group [21]. In clinical studies, the commercial Kyolic^®^ AGE has been successfully used to decrease the SBP, improve blood sugar regulation, IR, and cholesterol [22]. However, other results have described that garlic does not modify MS characteristics in human patients [10]. Despite this, other reports have shown that garlic and AGE may improve the independent pathologies comprising the syndrome such as hypertension, dyslipidemia, IR, and obesity. However, these altered variables are not isolated in the MS, being thus inter-related and the effects of garlic under this condition needs to be further analyzed.

### 4.1. Biochemical Variables

Our results show that the AGE treatment in MS animals decreased serum TG, insulin levels, and HOMA index, which are markers of the MS. Several studies have shown that garlic reduces blood cholesterol, TG levels, and SBP in hypercholesterolemic rats [23]. In rats administered with a high dose of fructose orally, the intraperitoneal injection of aqueous garlic extract increased insulin sensitivity and improved IR. This was associated with a significant reduction of serum TG and uric acid [24]. ROS are generated in diabetes by glucose oxidation and this, together with a decline of endogenous antioxidants, can lead to damage to cellular organelles, and the development of IR [24]. Furthermore, AGE decreased cholesterol in the C and MS rats. This suggests that AGE is an anti-cholesterolemic agent. The different organ sulfur compounds of AGE interfere directly with cholesterol biosynthesis inhibiting the HMG-CoA liver reductase levels and this is reflected in the lowering of the serum cholesterol levels which contributes to decrease of SBP [23]. Several garlic preparations cause a small reduction in cholesterol, LDL-C, and TG levels when taken for three months or less [25].

There were no significant differences in the glucose concentrations in any of our experimental groups since our model has no alterations in glucose metabolism [3,19,26]. The sucrose fed rats develop hyperinsulinemia and IR and therefore the serum glucose concentration does not change. Glucose surplus serves as a substrate for TG synthesis, which is reflected in hypertriglyceridemia and obesity. Similar results were described by Reaven and Ho [27]. However, the constant consumption of high sucrose by the rat alters several variables that constitute the SM present in this model.

In previous reports, we found that MS rats have—in addition to hypertriglyceridemia, hypertension, and IR—increased visceral adipose tissue weight even if the body weight is not always increased [3,28]. Although in this paper we did not determine fat weight, which is a limitation of the study, the results support previous data from our group showing that there are high circulating levels of leptin, being this adipokine directly proportional to fat content. In addition, leptin concentration decreases with the AGE treatment in MS rats, possibly reflecting a decrease in adipose tissue weight. Thus, AGE might participate in the regulation of the adipose tissue, but more investigations would be necessary to elucidate the mechanism. Our results show that, in the MS model, there is leptin resistance as was previously reported [21]. Moreover, garlic and its components reduce adiposity and restore the leptin/adiponectin imbalances and 1,2-vinyldithiin, a lipophilic garlic component, decreases leptin levels in differentiated pre-adipocytes [29,30]. Increased plasma leptin levels found in obese individuals (particularly in those with visceral obesity) a condition which is similar to the increases of this adipokine found in our rats, have been positively correlated with endothelial dysfunction, IR, oxidative stress, and type-2 diabetes [30].

Hyperglycemia causes increased protein glycation and the formation of advanced glycation end products which underlie the complications of MS. Glycation is accompanied by metal-catalyzed oxidation of glucose and Amadori products to form free radicals capable of protein fragmentation [31]. Our results showed that AGE treatment in MS rats decreases the advanced glycation end product concentration. This suggests that compounds found in AGE such as *S*-allyl cysteine have an antiglycative effect and can inhibit the formation of advanced glycation end products [32].

### 4.2. Vascular Reactivity and Cardiovascular Function

In vascular tissue, membrane-associated NAD(P)H oxidase generates the majority of the ROS which mighty underlie the pathological processes associated with endothelial dysfunction and vascular remodeling in MS [33]. In our MS model, aortic arterial vasoreactivity is altered. There is oxidative stress-associated hyper contraction and hypo relaxation, which contribute to hypertension [3]. The AGE treatment improved the vascular responses by decreasing vasocontraction and favoring vasorelaxation. These results suggest that the AGE components improve vasoreactivity. Allicin, which is formed by the action of the enzyme alliinase on the substrate alliin [34], is rapidly metabolized in the liver and then transformed to vinyldithiins in serum [35]. Allicin, and its metabolites may normalize vascular function [36]. In the feline mesenteric vascular bed, allicin induces vasodilation independent of β-adrenoreceptor activation. In pulmonary vascular resistance, allicin increases vasodilatation through the activation of cyclooxygenase, which mediates K^+^ (ATP) channel activity and causes hyperpolarization in smooth muscle cells [36]. Relaxation of vascular smooth muscle cells lowers SBP. NO synthetized by endothelial nitric oxide synthase (eNOS) activates the guanylate cyclase thus mediating Ach-induced vasodilation. The lack of NO production by eNOS is the major cause of vascular dysfunction contributing to hypertension [37]. The AGE normalizes NO from endothelial cells by preventing the decline of the levels of the eNOS cofactor BH_4_ [38]. AGE can also enhance NO production through activation of the eNOS pathway. NO suppresses contraction and increases the relaxation in the blood vessels, lowering SBP [8]. In addition, an analysis of the amino acid content of garlic powder showed the presence of high levels of arginine, the substrate for eNOS to synthetize NO. Assuming that 50% of arginine from garlic is absorbed, 1 g of garlic powder could increase the blood circulating concentration of this amino acid by up 10%. This shows that garlic can increase eNOS activity in a dose dependent manner [39]. In RAW 264.7 macrophages stimulated with LPS, garlic extract increased the eNOS activity through an increase in the arginine content [40]. Our results show an increase in NO, NO_3_^−^, and NO_2_^−^ ratios and citrulline, suggesting that AGE may increase eNOS synthase favoring a normal vascular function in the MS rats [41]. Furthermore, our results suggest that the AGE components can enhance vasodilatation by several possible mechanisms.

The aorta, not only serves a passageway, but it also plays an important role in the modulation of left ventricular performance, myocardial perfusion, and arterial function for the entire cardiovascular system [42]. The NO that is synthesized in the endothelial cells of the aorta, regulates blood pressure, cell-cell contact, and proliferation of the heart cells [20]. We found that cardiac functioning is decreased in MS rats due to the disease and to natural aging. Altered hemodynamic conditions decrease the blood flow and the oxygen supply to the tissues promoting oxidative stress which damages organs such as the heart, kidney, and liver. However, the AGE treatment improved cardiac performance in both the C and MS groups. These results suggest that there is a greater bioavailability of NO with the AGE treatment that counteracts the effects of oxidative stress.

Recent research has found that AGE components improved vascular levels associated with cardio protection attributed to its active ingredient *S*-allylcysteine. This substance increased NO levels by stimulating eNOS expression [22]. In the isolated heart of MS animals, the cardiac improvement in performance was associated with a decrease of the vascular resistance [42]. Our results show that the AGE effects might be mediated by NO derived from eNOS activation in the heart. Thus, AGE might have an important role in the modulation of left ventricular function and myocardial perfusion [42]. In addition, another study showed that doxorubicin, an anthracycline antibiotic, causes arrhythmia, ventricular extrasystole, intraventricular blockade, and bradycardia in mice and that the treatment with AGE (1.5 mg/kg body weight, three times per week for 40 days) can prevent myocardial damage and LPO in the heart [43]. In Wistar albino rats, oral administration AGE 250 mg/kg daily for 30 days prevents oxidative stress associated with lower ultra-structural changes induced by myocardial ischemic reperfusion injury [44]. Another clinical study showed that AGE inhibits the progression of coronary artery calcification, associated with lowering homocysteine levels [5].

Another mechanistic explanation for the beneficial effect of dietary AGE on cardiovascular system is through of the production of hydrogen sulfide (H_2_S). H_2_S is a biological signaling molecule involved in vascular system functions. It has recently attracted extensive attention for its multiple physiological functions and its potential contribution to pathological states such as hypertension, stroke, and metabolic disorders, including obesity and diabetes. H_2_S also acts as an antioxidant with anti-atherogenic, anti-apoptotic, anti-inflammatory, anti-proliferative properties [45]. Moreover, our data on IR are consistent with data from studies conducted in adipocytes showing that the metabolic actions of H_2_S are produced by the upregulation of the insulin-signaling pathways through activating phosphoinositide 3-kinase (PI3K) which leads to increases in the phosphatidylinositol-3,4,5-trisphosphate (PIP3) levels, AKT phosphorylation, and glucose utilization [46].

Besides, H_2_S may activate eNOS and increase NO bioavailability [47]. Therefore, this data may explain in part our results on the increase of NO levels in the MS rats treated with AGE. It has been reported that H_2_S production can be stimulated by AGE. The active metabolite allicin, is readily degraded into organic diallylpolysulfides that are potent H_2_S donors in the presence of thiols [46,48].

### 4.3. Hypertension

Arterial pressure is the result of cardiac output and vascular resistance. An alteration in its regulation results in hypertension. It is associated with functional and structural cardiovascular abnormalities, including reduced arterial elasticity and left ventricular diastolic dysfunction [1,4]. Our results show that the AGE treatment decreases the SBP in MS rats. This suggests that AGE exhibits anti-hypertensive properties that may modulate the cardiovascular system as we have previously discussed. Several mechanisms of action for the organosulfur compounds in AGE having SBP-lowering properties have been postulated, including mediation of intracellular NO production [49]. Furthermore, the gamma-glutamyl-cysteine present in garlic can lower SBP by inhibiting the angiotensin-converting enzyme. It increased the heart eNOS activity in a fructose-fed rat model and reduced the activity of aortic NAD(P)H oxidase and LPO in plasma [33]. In hyperinsulinemic, hyperlipidemic and hypertensive rats that were fed with high fructose (60%), allicin lowered SBP, serum insulin, and TG levels [50]. Likewise, garlic supplements help in the treatment of uncontrolled hypertension, lowering SBP by about 10 mmHg, a level similar to that obtained by standard SBP medication [22]. In Dahl salt-sensitive rats, AGE prevented hypertension and the progression of decompensated left ventricular hypertrophy and fibrosis through the enhancement of NO production [51].

### 4.4. GPx Activity and GSH Concentration

Cells have developed antioxidant defenses that include the GPx which destroys H_2_O_2_ through oxidized GSH to protect themselves from toxic ROS. GSH possesses a catalytic core of selenium [5]. Our results show that GPx activity in MS + SS decreased and this may be caused by a selenium deficiency. The AGE treatment favors an increase of this enzyme activity in the MS rats. GPx activity can be inactivated by selenium deficiencies or in conditions of oxidative stress in which O_2_^−^can inhibit the peroxidative function of the enzyme [52]. Previous investigations from our group have described that the MS rat model resulting from 30% sucrose chronic consumption, courses with oxidative stress [3,53]. Besides, selenium might also be decreased in MS and CVD patients [54]. Symptoms accompanying selenium deficiency in humans and animals demonstrate that it is an essential micronutrient [55]. Selenium participates in the regulation of GPx, since it is inserted in its active site. Thus, a decrease in selenium levels, or its absence, can affect the expression and activity of this enzyme [52]. AGE contains high levels of selenium which might underlie an increased activity of this enzyme [5].

AGE can act through different mechanisms such as direct neutralization of free radicals, and reduction of the peroxide concentration and alterations in the GPx pathway. Furthermore, AGE administration for five days at a concentration of 100 mg/Kg was able to activate phosphorylated Nrf_2_ factor in homogenates of cerebral cortex. This factor is associated with increased transcription of the genes for GPx and GST [56]. Besides being the substrate GPx, GSH itself is an important antioxidant low molecular weight molecule. It is constituted by cysteine, glutamate, and glycine. This tripeptide is the most abundant endogenous intracellular antioxidant present within cells and it may inactivate O_2_^−^and OH^−^ [57]. Approximately 85% is in a free form and the rest is bound to proteins. It may inactivate O_2_^−^and OH^−^radicals and it regenerates vitamins E and C, transforming them into their active forms [58]. In MS, reduced cellular and plasma levels of GSH are an indicator of oxidative stress [59]. Our results show that GSH concentration was significantly decreased in MS + SS, but our treatment restored it. A deficiency of GSH precursor amino acids such as cysteine, glutamate, and glycine could be the cause of GSH deficiency in MS. Furthermore, garlic contains unique organosulfur, *S*-allylcysteine, *S*-allylmercaptocysteine, *N*-fructosyl glutamate, *N*-fructosyl arginine, GSH, and selenium which have antioxidant capacities [6]. Thus, AGE can provide the essential amino acids that contribute to de novo generation of GSH or to the GSH level restoration itself [5]. In addition, AGE in cell culture prevented endothelial cells from oxidative stress by increasing cellular concentration of thiol antioxidants, such as cysteine and GSH [38]. GSH depletion compromises endothelial cell defenses against oxidative damage and may lead to cell death. Therefore, AGE can prevent intracellular GSH depletion and modulate the GSH redox cycle [60].

### 4.5. Lipid Peroxidation

Lipid peroxidation is a marker of damage by free radicals to the cell membranes. Our results show that LPO is increased in MS rats as a result of oxidative stress due to the high consumption of sucrose [3,54]. However, AGE exhibits antioxidant properties through the scavenger properties for OH^−^ and ^1^O_2_ of *S*-allylmercaptocysteine. Allicin scavenges OH^−^, O_2_^−^, and H_2_O_2_ and inhibits LPO. *N*α-(1-deoxy-d-fructos-1-yl)-l-arginine scavenges H_2_O_2_ [57]. Therefore, the results suggest that the increase in the concentration of GSH, GPx, and NO could partly explain the lowering of LPO observed in the MS rats treated with AGE. Likewise, a study showed that a raw garlic homogenate given for eight weeks, normalized both the increased LPO and GSH levels in liver in male Sprague Dawley rats fed with a high fructose diet (65%) [60]. Another study showed that AGE inhibits LPO in vascular endothelial cells induced by both H_2_O_2_ and oxidized LDL [24]. Our results are in accordance with previous findings that show the cardio protective effects of AGE and this may be related with reduction in oxidative stress as reflected in reduced LPO levels [61].

## 5. Conclusions

Our results show that the antioxidant properties conferred by the AGE treatment reduce the alterations in several variables that comprise the MS and its associated CVD-induced risk in a rat model of high sucrose fed animals. The anti-oxidant properties of AGE have already been proposed as mechanisms for the improvement of MS-unrelated hypertension and CVD.

## Figures and Tables

**Table 1:** General characteristics and serum biochemical values in the experimental groups. Abbreviations: C + SS = Control plus saline solution, C + AGE = Control plus aged garlic extracts, MS + SS = Metabolic syndrome plus saline solution, MS + AGE = Metabolic syndrome plus aged garlic extracts, SBP = Systolic Blood Pressure, TG = triglycerides, NO_3_^−^/NO_2_^−^ = nitrate and nitrite ratio, NO = nitric oxide. Data show the mean ± SEM, *n* = 8. C + SS and MS + Garlic vs. MS + SS ^†^ *p* < 0.05, * *p* = 0.01 and ** *p* = 0.001. C + SS vs. C + AGE * *p* = 0.01 and ** *p* = 0.001.

Variables	C + SS	C + AGE	MS + SS	MS + AGE
SBP (mmHg)	122.3 ± 4.2	122.6 ± 1.5	142.0 ± 2.8 *	127.9 ± 3.0 *
Glucose (mmol/L)	7.1 ± 0.5	6.1 ± 0.4	6.5 ± 0.1	6.4 ± 0.4
TG (mg/dL)	82.0 ± 6.9	67.1 ± 7.4	121.2 ± 4.7 **	93.7 ± 7.9 *
Cholesterol (mg/dL)	40.4 ± 3.0	33.7 ± 1.8 *	44.1 ± 1.2	35.2 ± 1.7 *
Insulin (µU/mL)	5.2 ± 0.6	6.2 ± 1.2	7.2 ± 0.3 ^†^	5.7 ± 0.5 ^†^
HOMA index	1.5 ± 0.2	1.6 ± 0.3	2.2 ± 0.1 ^†^	1.5 ± 0.1 ^†^
Leptin (ng/mL)	10.3 ± 0.7	11.1 ± 1.1	17.8 ± 1.3 **	12.4 ± 1.4 **
Advanced glycation end products (µU/mL)	420.8 ± 42.7	458.0 ± 67.8	1100.4 ± 103.9 **	643.0 ± 110.7 ^†^
Produts of the nitric oxide pathway				
Citrulline (µmol/L)	67.5 ± 6.1	77.9 ± 6.2	40.5 ± 5.2 **	74.6 ± 5.7 **
NO_3_^−^/NO_2_^−^ (µg/mL serum)	25.6 ± 1.5	26.6 ± 2.4	21.2 ± 0.8 *	24.9 ± 1.1 *
NO (pmol/mL perfusion liquid)	20.5 ± 1.3	30.2 ± 1.9 **	17.1 ± 0.5 *	27.7 ± 8.1 **
